# The Dry Revolution: Evaluation of Three Different EEG Dry Electrode Types in Terms of Signal Spectral Features, Mental States Classification and Usability

**DOI:** 10.3390/s19061365

**Published:** 2019-03-19

**Authors:** Gianluca Di Flumeri, Pietro Aricò, Gianluca Borghini, Nicolina Sciaraffa, Antonello Di Florio, Fabio Babiloni

**Affiliations:** 1 Department of Molecular Medicine, Sapienza University of Rome, Piazzale Aldo Moro, 5, 00185 Rome, Italy; pietro.arico@uniroma1.it (P.A.); gianluca.borghini@uniroma1.it (G.B.); fabio.babiloni@uniroma1.it (F.B.); 2 BrainSigns srl, via Sesto Celere, 00152 Rome, Italy; nicolina.sciaraffa@brainsigns.com (N.S.); antonello.diflorio@brainsigns.com (A.D.F.); 3 IRCCS Fondazione Santa Lucia, Neuroelectrical Imaging and BCI Lab, Via Ardeatina, 306, 00179 Rome, Italy; 4 Department Anatomical, Histological, Forensic & Orthopedic Sciences, Sapienza University of Rome, Piazzale Aldo Moro, 5, 00185 Rome, Italy; 5 College of Computer Science and Technology, Hangzhou Dianzi University, Hangzhou 310005, China

**Keywords:** brain activity, electroencephalography, wet electrodes, dry electrodes, frequency domain, power spectral density, machine-learning, wearable devices, mental workload

## Abstract

One century after the first recording of human electroencephalographic (EEG) signals, EEG has become one of the most used neuroimaging techniques. The medical devices industry is now able to produce small and reliable EEG systems, enabling a wide variety of applications also with no-clinical aims, providing a powerful tool to neuroscientific research. However, these systems still suffer from a critical limitation, consisting in the use of wet electrodes, that are uncomfortable and require expertise to install and time from the user. In this context, dozens of different concepts of EEG dry electrodes have been recently developed, and there is the common opinion that they are reaching traditional wet electrodes quality standards. However, although many papers have tried to validate them in terms of signal quality and usability, a comprehensive comparison of different dry electrode types from multiple points of view is still missing. The present work proposes a comparison of three different dry electrode types, selected among the main solutions at present, against wet electrodes, taking into account several aspects, both in terms of signal quality and usability. In particular, the three types consisted in gold-coated single pin, multiple pins and solid-gel electrodes. The results confirmed the great standards achieved by dry electrode industry, since it was possible to obtain results comparable to wet electrodes in terms of signals spectra and mental states classification, but at the same time drastically reducing the time of montage and enhancing the comfort. In particular, multiple-pins and solid-gel electrodes overcome gold-coated single-pin-based ones in terms of comfort.

## 1. Introduction

Almost a century ago (1924), the German physiologist and psychiatrist Hans Berger (1873–1941) was able to record human brain electrical activity through two electrodes placed on the scalp, applying for the first time techniques investigated some years before by his colleagues on animals [1]. This technique, that Berger named “electroencephalography” (EEG), is considered as “one of the most surprising, remarkable, and momentous developments in the history of clinical neurology” [2].

Nowadays, EEG is actually one of the most common and used techniques of neuroimaging, since compared to other techniques such as functional Magnetic Resonance Imaging (fMRI), Magnetoencelography (MEG) and functional Near-InfraRed spectroscopy (fNIRS), it is able to ensure at the same time great temporal and potentially good spatial resolution (with a high number of electrodes), it is relatively cheap and portable [3]. For such a reason, during the last decades EEG has gone beyond hospital doors, becoming not only a reliable monitoring system for healthcare [4], but also a powerful and versatile tool for investigating human brain physiology, cognition and behavior [5,6]. Because of the increasing interest and demand of such technology, and thanks to the fast progresses of micro- and nanoelectronic industry, biomedical devices manufacturers have been able to drastically reduce the dimensions of EEG devices. Therefore, if in the 60s an EEG device required a room and meters of cables, modern EEG devices are no larger than a smartphone and equipped with storage support, long-life batteries and wireless communication. These great improvements are at the basis of the recent successes of cognitive and behavioral neuroscience research, since it is now possible to investigate human brain physiological dynamics while working or performing everydayness activities, such as driving the car [7,8,9] or piloting an aircraft [10,11,12], working in control rooms [13,14,15,16], watching the TV [17,18], doing sport [19], eating [20], listening music [21,22] or smelling fragrances [23,24]. EEG devices are now wearable and they are considered the key tool to bring research about Brain-Computer Interfaces (BCI, i.e., those system aimed to adapt the objects behavior on the basis of current human mental states) from the laboratory to the applicative fields [25,26]. In this context, several prototypes of EEG-based BCIs have been already validated in real settings [27,28], however a still present issue limiting the development and employment of such technologies is their ease of use and acceptability to the user.

In fact, despite the great improvements in terms of EEG device sizes and features, the quality and reliability of this technology still relies on recording brain signals through metallic sensors to be applied over the rubbed scalp and by adapting impedances through gels or other conductive substances. More in detail, EEG electrodes are produced with the shape of a cup, disc or needle, and are usually made of silver (Ag) and silver chloride (AgCl) [29]. Because Ag is a slightly soluble salt, AgCl quickly saturates and comes to equilibrium. Therefore, Ag is a good metal for metallic skin-surface electrodes [30]. Before putting the electrodes over the scalp, it is necessary to rub scalp skin with pastes or alcohol solutions, in order to remove all the substances and any kind of impurity generally present over the scalp epidermis. Then, traditional Ag/AgCl electrodes require electrolyte gel that facilitates the transduction of the ionic currents between the skin and the electrode. Furthermore, the electrode-skin impedance must be controlled and adapted to achieve acceptable low values, typically 5 ÷ 20 KΩ [31,32]. These are mainly manual actions that require technicians with expertise in EEG recordings. Another remarkable inconvenience is the annoyance caused to the subject under test. For instance, the abrasive paste and the electrolyte gel, despite being minimally invasive and barely harmful, are sticky products that make the hair and scalp wet and dirty. Also, the time needed to adapt the impedance can last long. The use of a massive electrolyte to speed up the impedance adaptation could cause electrical bridges between electrodes, thus being counterproductive. Last but not least, once acceptable impedances values have been achieved, the following issue will be gel drying, thus causing degradation of its conductive properties. For example, Lin and colleagues [33] measured an impedance deterioration of wet electrodes from 5 to 15 kΩ within 5 h after gel application. Many of these problems can be minimized by using dry electrode systems [34], therefore the research about EEG dry electrodes, started during the nineties [35,36], is recently living a very fertile period. Let us think that some years ago practical dry electrodes have been identified as one of the two disruptive technologies in BCI research [37,38].

Recent studies produced a wide variety of EEG electrode concepts based on dry technology, including silicone conductive rubber [39], comb-like and multi-pin electrodes [40,41], gold-plated electrodes [37,42], bristle-type electrodes [43], and foam-based sensors [33]. In general, all these solutions could be categorized into spiky, capacitive/non-contact or other heterogeneous types of contact [44]:

- Spiky Contact: in this solution, the electrode surface consists of linear or circular array of spikes that come into direct contact with the scalp;
- Capacitive/Non-contact: since the absence of impedance adaptation substances could make the skin-electrode contact instable over time, some researchers coped with this difficulty by avoiding physical contact with the scalp through non-conductive materials (i.e., a small dielectric between the skin and the electrode itself): despite the extraordinary increase of electrode impedance, in this way it will be quantifiable and stable over time [45];
- Others: other heterogeneous approaches in terms of materials, such as foams or solid gels [46].

In addition, because of their intrinsic higher impedance, if compared with traditional wet electrodes, a complementary solution could be to on-site amplify (i.e., just over the electrode, and upstream the cable toward the device amplifier) the signal with ultra-high input impedance amplifiers (the so-called “active electrodes”).

Several attempts are already present in literature about the comparison and validation of these innovative dry electrodes [42,47]: there is the common opinion that wet electrodes have to be still considered the gold standard [42,44], however the gap between wet and dry electrodes is more and more reduced. Of course, it is important to take into account that low-cost devices (i.e., a few hundred euros, such as Emotiv EPOC or Neurosky MindWave) are still far from being reliable for applications other than gaming and playful activities in general [48,49]. Nevertheless, in their recent review [44] Lopez-Gordo and colleagues pointed out that, among this wide variety of works, there is still the lack of a comprehensive comparison between different electrodes concepts. In fact, the works are generally focused on contrasting a specific dry solution versus traditional wet electrodes, or investigated a specific application (e.g., only ERPs): sometimes they are based just on qualitative analysis while only few studies report some statistics, such as correlation or specific signals spectral features. They concluded that “different dry electrode approaches are conceptually distinct and, in the literature, reports of performance have been carried out with non-homogenous methodologies, so their results cannot easily be discussed or compared”, thus encouraging additional work to evaluate the level of maturity achieved by dry technology for the recording of EEG signals in clinical and other applications [44].

In this context, the present work aims to provide a comprehensive comparison between three different types of dry EEG electrodes, among them and with respect to wet electrodes. The wet electrodes consisted in traditional Ag/AgCl ring-shaped electrodes, while the three dry types consisted in:

- Active gold-coated single pin electrode;
- Hybrid (capacitive/conductive) multiple-spikes based electrode;
- Passive solid-gel based electrode.

The three different solutions of EEG dry electrodes have been selected in order to provide a comprehensive state-of-art contribute. In fact, all the electrodes employed within this experiment: (a) have been recently produced by leading companies of this domain; (b) are both passive and active; and (c) provide three different types in terms of electrode material and shape. A comparison has been made taking into account different factors, following the suggestions of Lopez-Gordo and colleagues [44]: subjective spectral features (i.e., the Individual Alpha Frequency during a rest closed eyes condition [50]), signal power spectra correlations during closed and open eyes conditions, mental states classification performance in terms of mental workload, comfort perceived by the user and easiness of use for the operator.

## 2. Materials and Methods

### 2.1. Experimental Protocol

Twelve healthy subjects (29.7 ± 3.9 years old), all males and recruited on a voluntary basis, participated to the experiment. Their hair length ranged from 0 (i.e., bald) to 8 cm. Informed consent for both study participation and publication of pictures was obtained from all the subjects after the explanation of the study. The experiment was conducted following the principles outlined in the Declaration of Helsinki of 1975, as revised in 2000. The study protocol received the favourable opinion and approval by the Ethical Committee of the Sapienza University of Rome. Only aggregate information has been released while no individual information was or will be diffused in any form.

The experimental protocol consisted in repeating four times a sequence of tasks, one time per each electrode type (i.e., traditional WET Ag/AgCl, ACTIVE DRY SINGLE-GOLD-PIN-based, HYBRID DRY MULTIPE-SPIKES-based and PASSIVE SOLID-GEL-based electrodes, see following paragraph for further details). The tasks sequence consisted in two rest conditions, 1 min with Closed Eyes (CE) and 1 min with Open Eyes (OE), followed by two 3-min-long mathematical tasks. In particular, the two rest conditions (i.e., CE and OE) have been chosen in order to not elicit any specific brain activity in the subjects and perform comparisons among the different electrode types in terms of signal spectral features [44]. During the CE condition the subject was asked to stay relaxed, whereas during the OE condition to fix a point on the screen, specifically a white cross on a black background. Instead, the two mathematical tasks have been chosen in order to elicit two different levels of mental workload and to compare the signal recorded by means of the different electrode types in terms of classification performance. In particular, the mathematical task consisted in solving repeated additions proposed to the subjects through a desktop computer: each subject was asked to solve the additions trying to achieve his best performance (i.e., to provide the correct answer within the least time possible). The two levels of task difficulty (to elicit two different levels of mental workload) were designed accordingly to the principles adopted by Zarjam and colleagues [51]. More in detail, the EASY task consisted in a 1- and 2-digits numbers without any carry sum (e.g., 5 + 54, *Very low* level in [51]), while the HARD task consisted in a 2- and 3-digits numbers with 1 carry sum (e.g., 31 + 477, *High* level in [51]). This task was chosen because there is a rich literature on the concepts and procedure of mental arithmetic operations [52], while in [53] it is shown that the manipulation of the number of carry operations and the value of the carry is an important variable in varying the difficulty of arithmetic sums. Each subject took confidence with the mathematical task before the beginning of the experiment, in order to avoid any eventual training effect [54,55], while among the four repetitions each subject started two times with the Easy and two times with the Hard task in a randomized way, in order to avoid any habituation effect.

In addition, the order of the four repetitions (one per each electrode type) of the afore-described tasks sequence was randomized among subjects in twelve different combinations in a balanced way, i.e., each electrode type has been tested three times as the first, three as the second, three as the third, and three as the fourth and last one. It is important to consider that after the test of the wet sensors, the subject’s hairs were carefully washed in order to remove any gel residual before testing the next scheduled system.

**Figure 1:**
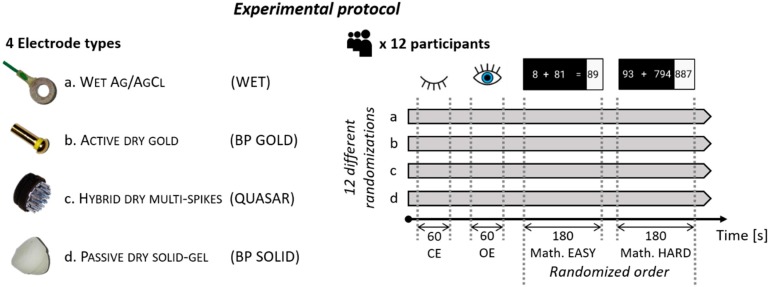
Schematic summary of the experimental protocol, with a picture of each electrode employed during the experiments.

At the end of each repetition the subject was asked to evaluate on a 0-to-10 Visual Analogue Scale (VAS) the comfort (where 0 stood for discomfort/complaint, while 10 for maximum comfort) of that specific EEG device, while at the end of the whole experimental protocol he was asked to rank the devices in terms of comfort itself. Furthermore, the time of montage of each EEG device for each subject was gathered. Figure 1 represents a schematic summary of the experimental design, including a picture of the four electrodes employed during this work.

### 2.2. The four EEG Electrode Types

The three different solutions of EEG dry electrodes have been selected in order to provide a comprehensive state-of-art contribute (please refer to the Introduction). In fact, all the electrodes employed within this experiment: (i) have been recently produced by leading companies of this domain; (ii) are not employed by low-cost market sector (because of the intrinsic poor signal quality of low-cost devices [49]); (iii) are both passive and active systems; and (iv) provide three different types in terms of electrode material and shape. This manuscript does not aim at providing technical focuses on EEG electrodes composition, however a brief description of each one of them (represented in Figure 1) is provided in the following paragraphs in order to differentiate them in terms of key features.

#### 2.2.1. Traditional Wet Ag/AgCl Electrodes (Wet)

The wet electrodes employed during the experiments were traditional ring-shaped Ag/AgCl electrodes (electrode ‘a’ in Figure 1), produced by EasyCap GmbH (Herrsching, Germany [56]), connected through silver wires to the amplifier. In particular, in terms of EEG amplifier, the BeMicro device (EBNeuro, Firenze, Italy [57]) was employed. The signals have been acquired without any hardware filter at a sampling frequency of 256 Hz. For the impedances adaptation, it has been used the electroconductive gel “ELGEL-P” produced by SEI EMG srl (Cittadella, Italy [58]). They will be hereinafter labelled “Wet”.

#### 2.2.2. Active Dry Single Gold Pin-Based Electrodes (BP Gold)

In this case, the electrodes consisted in a gold-coated single pin with the shape of a mushroom (electrode ‘b’ in Figure 1). They are produced in 3 different lengths (10, 12 and 14 mm) to choose depending on the scalp site and subject hairs. The signal is amplified just after the electrode (gain factor = 1, Input impedance > 200 MΩ). These electrodes, named actiCAP Xpress QuickBits, are produced by BrainProducts GmbH (Gilching, Germany [59]), and the signals have been acquired at a sampling frequency of 250 Hz through the LiveAmp device [60] produced by BrainProducts itself. They will be hereinafter labelled “BP Gold”.

#### 2.2.3. Hybrid Dry Multiple Spikes-Based Electrodes (Quasar)

In this case, the electrodes consisted in hybrid biosensors using a combination of high impedance resistive and capacitive contact to the scalp [61]. Electrical contact is made through two rings of spikes, with a total diameter of about 3 cm (electrode ‘c’ in Figure 1). The amplifier electronics are shielded and mounted immediately behind the electrode (Input impedance > 10 GΩ) in order to limit interference caused by external signals. They are produced by Quasar Inc. (San Diego, CA, USA [62]). The signals have been acquired at a sampling frequency of 300 Hz through the related DSI-7 device [63] produced by Wearable Sensing LLC (San Diego, CA, USA [64]). They will be hereinafter labelled “Quasar”.

#### 2.2.4. Passive Dry Solid-Gel Based Electrodes (BP Solid)

In this case, the electrodes consisted in a cone made of a hygroscopic solid gel (electrode ‘d’ in Figure 1). They have to be kept immersed in a saline solution for a few minutes before the experiments, in order to be hydrated, and then to keep their characteristics stable for almost 8 h. They are a prototype (thus still not available on the market) distributed by BrainProducts GmbH [59]. The signals have been acquired at a sampling frequency of 250 Hz through the LiveAmp device [60] produced by BrainProducts itself. They will be hereinafter labelled “BP Solid”.

### 2.3. Data Acquisition and Processing

The EEG montage, in terms of electrodes position and referencing, was chosen in order to employ a common design among the four devices. For such a reason, EEG data were acquired from six channels, placed accordingly to the 10–20 International Standard: F3, F4, C3, C4, P3 and P4. Depending on the device, all the channels were referred physically or digitally to Pz, while the ground electrode was placed over the FCz position.

**Figure 2:**
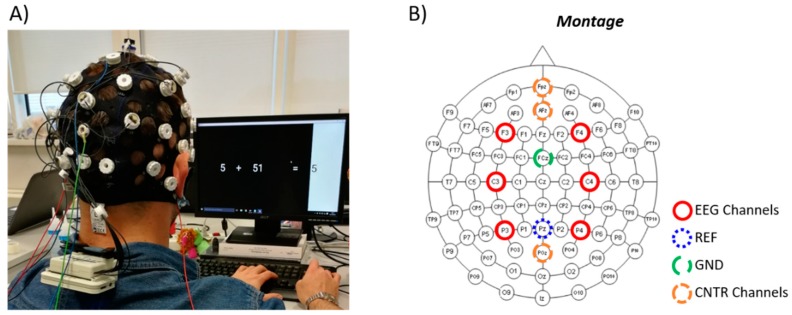
(**A**) Moment of the experimental protocol during the mathematical task, in particular the EASY one during the testing of active dry gold electrodes. (**B**) Graphical representation of all the electrodes position over the scalp.

At this point, the results could depend on two main factors: the different electrode type, and the different recording moment (the electrode types were tested consecutively and not simultaneously). Ideally, the maximum result for all the performed analysis would be achieved if during the four repetitions the electrodes were not changed. Nevertheless, there is a certain bias while comparing on the same subject the same conditions recorded in different times. For such a reason, three additional EEG channels were placed and kept fixed during the whole experiment (thus during each electrode type testing) over Fpz, AFz and POz. In this case, traditional wet electrodes were used, and they will be hereinafter labelled “*Control*” electrodes (please see Figure 2B for the graphical representation of all the electrodes position over the scalp). The aim of their employment was to separate these two factors (electrode-related effect and time-related effect), since the Control (CNTR) electrodes were not changed while the different electrode types were tested.

All the data were recorded in raw format and were offline processed by using Matlab software (MathWorks Inc., Natick, MA, USA). More in detail, the acquired signals were band-pass filtered by a 5th order Butterworth filter (low-pass filter cut-off frequency: 40 Hz, high-pass filter cut-off frequency: 1 Hz). Additionally, a notch filter was applied in order to remove any eventual interference of the mains frequency (50 Hz). The dataset was then segmented in 2-s-long epochs, shifted of 0.5 s, with the aim to have both a high number of observations in comparison with the number of variables, and to respect the condition of stationarity of the EEG signal [65]. In fact, this is a necessary assumption in order to proceed with the spectral analysis of the signal. For other sources of artefacts, specific procedures of the EEGLAB toolbox were applied [66]. In particular, three methods were used: a *threshold-based*, a *trend estimation-based* and *a sample-to-sample difference-based* criterion [67]. In the threshold criterion an epoch was marked as “artefact” if the signal amplitude was higher than ±80 μV. In the trend estimation the epoch has been interpolated in order to check the slope of the trend within the considered epoch. If such slope was higher than ±10 µV per second, the considered epoch was marked as “artefact”. Finally, the sample-to-sample difference was calculated: if such a difference, in terms of absolute amplitude, was higher than 25 μV, i.e., an abrupt variation (no-physiological) happened, the epoch was marked as “artefact”. At the end, the EEG epochs marked as “artefact” were removed from the EEG dataset with the aim to obtain an artifact-free dataset from which estimating the parameters for the various analyses. The Power Spectral Density (PSD) was then estimated by using the Fast Fourier Transform (FFT) on the *artifact-free* dataset with 2 s-long Hanning windows, therefore a frequency resolution of 0.5 (Hz) was achieved.

### 2.4. Performed Analysis

#### 2.4.1. Evaluation of the “Time Effect” on the Recordings

As described before, the four EEG devices were not used simultaneously but in a consecutive way. In literature, it is argued that such approach could induce a certain bias, since it is not implicit the comparability of two equal conditions recorded in two different times, although being rest conditions of the same subject [44]. However, also the setup based on simultaneous recording from contiguous electrodes is debated. In fact, on one hand very close electrodes could lead to electrical bridges caused by the spreading of gel on the skin surface. It has been demonstrated that an average spread of 1 cm in each direction under the scalp is typical during EEG recordings, thus a separation between electrodes of more than at least 2 cm is suggested [43]. But, on the other hand, the higher such separation is, the higher the probability of recording different electrical activity will be.

Therefore, in this study a validation of the experimental hypothesis by evaluating the “time effect” has been performed before going through the planned analysis. This kind of evaluation was performed by employing data recorded through the Control (CNTR) electrodes.

Taking into account that traditional wet electrodes were employed and that they were kept fixed during the whole experiment, the brain activity recorded on each electrode during the four repetitions was compared in terms of correlation. In particular, the Pearson’s correlation coefficient has been estimated for each subject for each electrode, and during both the rest conditions (OE and CE), between the PSD values of the first repetition and those of the three following ones, independently from the electrode type tested during that specific repetition. The PSD values ranged from 2 to 35 Hz (with a frequency resolution of 0.5 Hz, the vectors are 67 points long). The distance between each repetition was equal to 25 min. All the comparisons have been analyzed through multiple repeated measures ANOVAs, and in case of significance, by performing Duncan’s post-hoc tests.

#### 2.4.2. IAF Estimation

The four electrode types were firstly compared in terms of Individual Alpha Frequency (IAF) estimation. In fact, Klimesch [50] demonstrated that subjective alpha frequency range varies to a large extent as a function of age, neurological diseases, memory performance, brain volume and task demands, therefore the use of fixed frequency bands does not seem justified. For such a reason, recent works investigating brain activity use to define subjective EEG bands as a function of the IAF [55,68,69,70,71,72]. In particular, accordingly to the method suggested by Klimesch and colleagues [73], the IAF has been estimated for each subject for each EEG device (i.e., the four electrode types) as the peak between 7.5 and 12.5 Hz of the PSD averaged over the parietal channels during the Closed Eyes condition.

A repeated measures ANOVA has been performed on the obtained IAF values in order to investigate eventual differences with respect to the traditional wet electrodes. Post-hoc analysis has been then performed by the Duncan test. Also, the Mean Squared Error (MSE) has been calculated between the IAF values estimated while using the three dry electrodes types and the traditional wet ones.

#### 2.4.3. Power Spectra Comparison

The power spectral densities (PSD) of the EEG signals recorded through the three dry electrode types were compared by means of the correlation, for each channel and subject, with the PSD of the signals recorded through the traditional wet electrodes. For this kind of analysis only data related to the Open (OE) and Closed Eyes (CE) conditions have been used, since EEG signal can be considered stationary for prolonged time (i.e., more than few seconds) only during resting states, while the mathematical tasks were designed in order to elicit particular cognitive processes [74].

More in detail, the Pearson’s correlation coefficient was calculated between the signal power spectra of a specific dry electrode type and of the traditional wet one in a coherently way, i.e., for each channel for each condition and for each subject. Let us make a practical example: the power spectrum of the EEG signal recorded through the *BP Solid* electrode over the F3 position, during the OE condition of Subject X, has been correlated with the power spectrum of the signal recorded through the traditional wet electrode over the F3 position during the OE condition of the same Subject X. In addition, for this kind of analysis the behavior of “*Control*” electrodes has been also investigated (please refer to Par. 2.3). In particular, they remained in their positions during the test of all the devices. Taking into account that the same electrodes used as “traditional wet electrodes” were employed, the PSD values of their signals recorded during the three dry electrodes types have been averaged and correlated with the PSD values obtained during the use of wet electrodes, in order to estimate the maximum correlation achievable if the electrodes type was not changed during tasks repetition. However, they have been used only for qualitative analysis, since their position was different from the position of tested electrodes.

The global correlation has been computed averaging, per each device (i.e., the four electrode types), the PSD values ranging from 2 to 35 Hz (with a frequency resolution of 0.5 Hz, the vectors are 67 points long) over the six investigated channels. In addition, in order to evaluate any eventual effect of the electrode position as well as of the frequency dependence, the correlation has been also investigated channel by channel and dividing the whole spectrum in three sub-bands of equal length:

- From 3.5 to 12 Hz (18 points), approximately coinciding with Theta and Alpha rhythms;
- From 12.5 to 21 Hz (18 points), approximately coinciding with lower Beta rhythms;
- From 21.5 to 30 Hz (18 points), approximately coinciding with higher Beta rhythms.

The sub-bands choice was made on the basis of a compromise between (i) having bands not too short (the samples size will impact on the correlation robustness); and (ii) having bands of the same length (in order to make possible the comparison among them).

All the comparisons were analyzed through multiple repeated measures ANOVAs, and in case of significance, by performing Duncan’s post-hoc tests.

#### 2.4.4. Mental States Classifier Performance

In order to evaluate also the suitability of such sensors for passive BCI-based applications [26], a simple classification performance analysis was performed. For this aim, the EEG data recorded while performing the two mathematical tasks (EASY and HARD) were used. Among the wide range of machine-learning algorithms, the StepWise Linear Discriminant Analysis (SWLDA) [75] was employed. Since the purpose of this manuscript is not to evaluate the performance of different algorithms but only the impact of the different EEG electrodes on this kind of applications, such algorithm was chosen because of its proved reliability for human workload classification [67,76,77,78].

For each subject for each EEG device, the EEG data of each mathematical task have been divided in a 2-min-long segment to use as “Training dataset” and the remaining 1-min-long segment to use as “Testing dataset”. Two different combinations have been realized: (1) TRAINING equal to the first 2 min and TESTING equal to the last one; (2) TRAINING equal to the last 2 min and TESTING equal to the first one. In particular, for each subject the features domain consisted of the EEG PSD values of the frontal Theta ([3.5 ÷ 7.5] Hz) and parietal Alpha ([4 ÷ 8] Hz) rhythms, since they are considered and demonstrated to be the more relevant brain activity features related to mental workload [79,80].

Therefore, the analysis of the Area Under the Curve (AUC) of the Receiver Operator Characteristic (ROC) curve of the classifier was performed [81]. In fact, AUC represents a widely used methodology to test the performance of a binary classifier: the classification performance can be considered good with an AUC higher than at least 0.7 [82]. In this case, there are actually two classes in terms of mental workload, i.e., Easy and Hard, related to the two different difficulty levels of the mathematical task. For each subject for each device, the AUC values of combinations 1 and 2 were averaged. Finally, a repeated measures ANOVA was performed on the classification performance in order to investigate any eventual effect of the electrodes type.

#### 2.4.5. Usability

The usability of each electrode type was evaluated in terms of time necessary for the montage (that is intrinsically related to the time necessary to adapt the impedances) and comfort perceived by the subjects wearing the EEG device. In this regard, please consider that the EEG montage was done by two operators with comparable expertise and strong knowledge of the field, with hundreds of previous EEG recordings. In particular, the two factors were investigated as follows:

(a) Easiness to use: a repeated measures ANOVA was performed on the montage times;
(b) Comfort: a repeated measures ANOVA was performed on the comfort VAS scores assessed by the experimental subjects at the end of each repetition; also, the devices ranking was analyzed by an analysis of frequencies.

In case of significance, Duncan’s post-hoc tests were performed.

## 3. Results

### 3.1. Evaluation of the “Time Effect” on the Recordings

**Figure 3:**
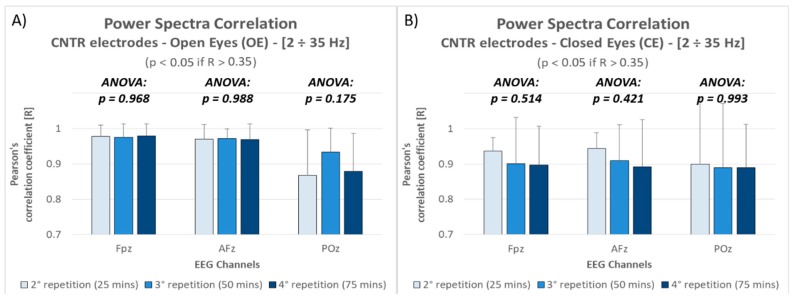
The bar graph represents the mean and the standard deviation of the Pearson’s correlation coefficients between the first and the three following repetitions, for each electrode position, during the Open Eyes (**A**) and Closed Eyes (**B**) conditions. The p-values of the ANOVAs performed for each comparison are reported within the graphs.

The analysis performed on the correlation between signals power spectra between the first and the three following repetitions of the Open and Closed Eyes tasks (Figure 3) revealed that:

- Independently from the channel and the repetition, all the recorded activities were positively and significantly (all *p* < 0.05) correlated with brain activity recorded at the beginning of the experiment;
- The correlation did not significantly change over time, i.e., from the first to the last repetition (approximately 75 min after the first one), as revealed by ANOVAs performed for each channel and for each condition.

### 3.2. IAF Estimation

**Figure 4:**
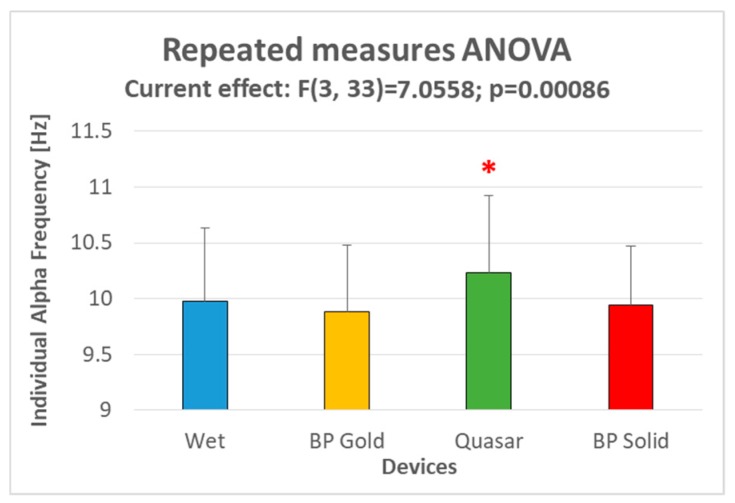
The bar graph represents the mean and the standard deviation of the IAF values estimated during the CE condition through the four different electrode types. The red asterisk indicates the sample significantly (*p* < 0.05) different from the other ones, as demonstrated by the Duncan’s post-hoc test.

The results in terms of IAF estimation are reported in Figure 4. The ANOVA revealed a significant effect (F = 7.06; *p* = 0.0009) related to the devices, i.e., the electrode types. In particular, the Duncan’s post-hoc test showed that the IAF values estimated through the Quasar electrodes were significantly different from those one estimated through the other electrode types (all the *p* < 0.05), while no differences arose among the latter ones. In terms of Mean Squared Error with respect to the wet electrodes, the IAF values differed of 0.1 Hz^2^ (BP Gold), 0.12 Hz^2^ (Quasar) and 0.09 Hz^2^ (BP Solid).

### 3.3. Power Spectra Comparison

**Figure 5:**
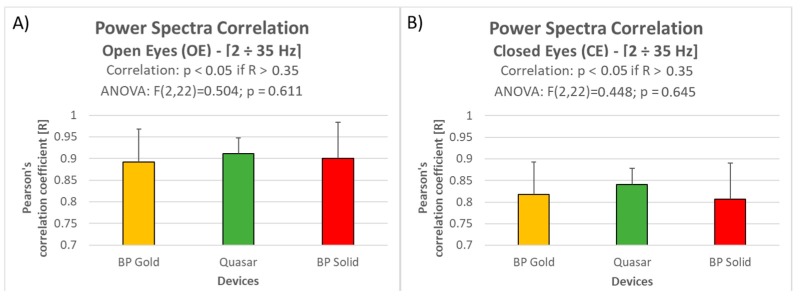
The bar graphs represent the mean and the standard deviation of the Pearson’s correlation coefficients between the three dry electrode types and the traditional wet ones during the OE (**A**) and CE (**B**) conditions, including the repeated measures ANOVAs results.

The overall results in terms of spectra correlation during the Open and Closed Eyes conditions are reported in Figure 5. The power spectra of the EEG signals recorded through the three dry electrode types are all positively and significantly correlated with the power spectrum of the signals recorded through traditional wet electrodes. In fact, a correlation between two samples with a degree of freedom equal to 65 (two 67-points-long vectors, thus n-2 = 65) is considered significant (assuming a *p* < 0.05) if R (Pearson’s correlation coefficient) is higher than 0.24 [75]. Actually, in this study the correlations analysis is based on 12 different correlations per device (one for each subject), thus the significant p-value threshold has been corrected through the Bonferroni correction for multiple comparisons [83,84]:

α’ = α/*n*

where *α* is the targeted value of significancy (i.e., 0.05), *n* the number of multiple comparisons and *α’* the equivalent value (in our case α’ = 0.05/12 = 0.00415). Therefore, the significant R-threshold to consider, related to the corrected *p*-value, is 0.35. Also considering this new threshold after the multiple comparisons correction, the correlation are still significant.

Of course, the correlations could be not equal to 1 since the recording are not simultaneous but consecutive. In fact, the analysis over the CNTR electrodes (Figure 3) demonstrated as also by using the same electrodes, the maximum correlation achievable is close but less than 1. With respect to the Control electrodes, the signal recorded with the dry electrode types revealed a mean correlation decreasing of 5% and 9% during respectively Open and Closed Eyes conditions, however the ANOVAs did not show any significant difference among them (Open Eyes: F = 0.504; p = 0.611. Closed Eyes: F = 0.448; *p* = 0.645).

**Figure 6:**
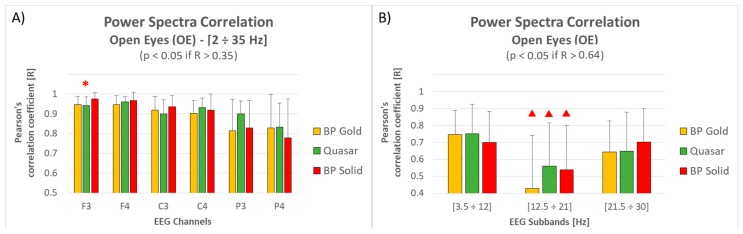
(**A**) The bar graph represents the mean and the standard deviation of the Pearson’s correlation coefficients between the three dry electrode types and the traditional wet ones with respect to the electrode position. The red asterisk indicates the comparison where the ANOVA revealed significant differences. (**B**) The bar graph represents the mean and the standard deviation of the Pearson’s correlation coefficients between the three dry electrode types and the traditional wet ones with respect to the frequency. The red triangle indicates the case of non-significant correlation (on average) with traditional wet electrodes.

Figure 6 shows how the electrodes position and frequency affect electrode performance during the OE condition. In particular:

- Figure 6A shows a correlation decreasing (BP Gold = −13%, Quasar = −9%, BP Solid = −17%) moving from frontal to parietal sites for all the electrode types, however the correlation values are still significant and high (>0.75 and thus higher than 0.35); the ANOVA performed over each electrode did not reveal any significant difference except than for F3, where the correlation of the BP Solid electrode was significantly higher than both the other two electrode types (Duncan test: *p* = 0.02 vs. BP Gold, *p* = 0.01 vs. Quasar);
- Figure 6B shows a correlation decreasing in correspondence of lower beta frequencies (between 12.5 and 21 Hz) for all the electrode types, without any significant difference among them for each sub-band. Also, considering that for this comparison the correlation degrees of freedom are equal to 16 (two 18-points-long vectors, thus n-2 = 16) and consequently the significant threshold R = 0.64 (taking into account the Bonferroni’s multiple comparisons correction), the three dry electrode types revealed a no-significant mean correlation in such sub-band.

**Figure 7:**
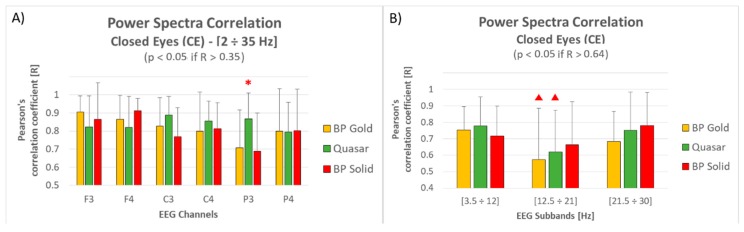
(**A**) The bar graph represents the mean and the standard deviation of the Pearson’s correlation coefficients between the three dry electrode types and the traditional wet ones with respect to the electrode position. The red asterisk indicates the comparison where the ANOVA revealed significant differences. (**B**) The bar graph represents the mean and the standard deviation of the Pearson’s correlation coefficients between the three dry electrode types and the traditional wet ones with respect to the frequency. The red triangle indicates the case of non-significant correlation (on average) with traditional wet electrodes.

Figure 7 shows how the electrodes position and frequency impact on electrode performance. In particular:

- Figure 7A shows a correlation decreasing moving from frontal to parietal sites for BP Gold (−15%) and BP Solid (−16%) electrode types, while Quasar type did not show any particular variation (≈1%). However, it is important to note that: (i) the correlation values over the frontal sites for BP Gold and BP solid electrodes were higher than Quasar electrodes; (ii) all the correlation values are still significant and high (>0.7, and thus higher than 0.35); (iii) the ANOVA performed over each electrode did not reveal any significant difference except than for P3, where the correlation of the Quasar electrode was significantly higher than both the other two electrode types (Duncan test: *p* = 0.017 vs. BP Gold, *p* = 0.014 vs. BP Solid);
- Figure 7B shows a correlation decreasing in correspondence of lower beta frequencies (between 12.5 and 21 Hz) for all the electrode types, without any significant difference among them for each sub-band. Also, considering that for this comparison the correlation degrees of freedom are equal to 16 (two 18-points-long vectors, thus n-2 = 16) and consequently the significant threshold R = 0.64, the correlation values of only BP Solid electrode type are still significant for each sub-band, while BP Gold and Quasar electrodes suffered a significant decreasing in correspondence of lower Beta frequencies.

### 3.4. Mental States Classification Performance

**Figure 8:**
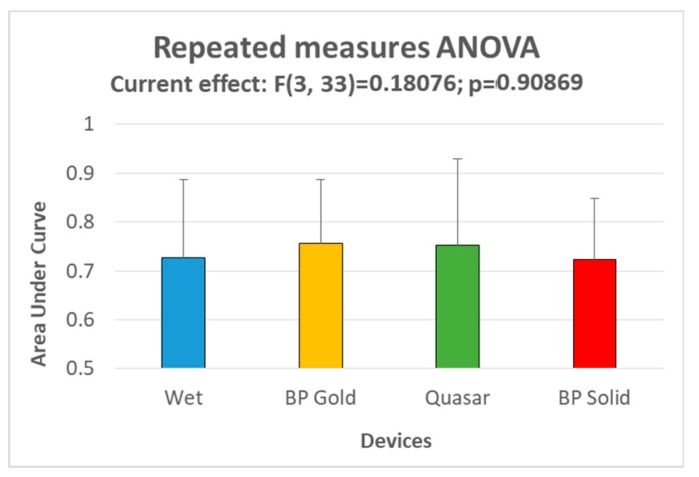
The bar graph represents the mean and the standard deviation of the Areas under Curve (AUC) of the Receiver Operator Characteristic (ROC) curve of the classifier while discriminating EASY and HARD workload levels during the mathematical task.

In terms of workload classification, it was possible to achieve good results of discriminability between the two classes (EASY and HARD) with all the three dry electrodes types (mean AUC: 0.75 for BP Gold, 0.75 for Quasar, 0.73 for BP Solid electrodes), not significantly different from the performance achieved through traditional wet electrodes, as showed by the Repeated measures ANOVA (F = 0.181; *p* = 0.909; Figure 8).

### 3.5. Usability

**Figure 9:**
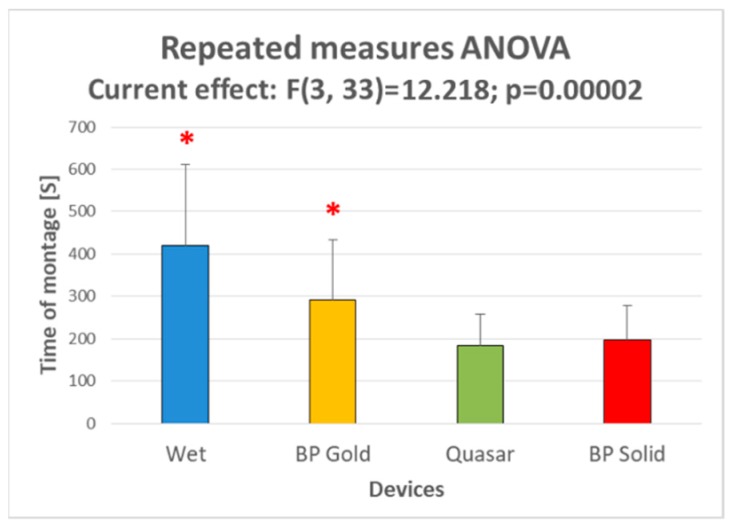
The bar graph represents the mean and the standard deviation of the times of montage of each device. The red asterisks indicate the samples significantly (*p* < 0.05) different from the other ones, as demonstrated by the Duncan’s post-hoc tests.

The ANOVA performed over the time of montage necessary for each type of devices (i.e., electrode types) showed a significant effect (Figure 9). In particular, the Duncan post-hoc tests showed that:

- Traditional wet electrodes required the longest times of montage (>5 min), significantly higher than all the dry types (*p* = 0.007 vs. BP Gold electrodes, *p* < 10^−4^ vs. Quasar and BP Solid electrodes);
- BP Gold electrodes required a time of montage (≈5 min) significantly lower than traditional wet electrodes but higher than Quasar (*p* = 0.026) and BP Gold electrodes (p = 0.04);
- Quasar and BP Solid electrode types required the shortest time of montage (≈3 min), without any significant difference among them.

**Figure 10:**
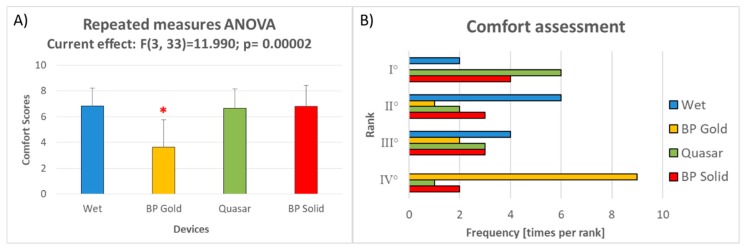
(**A**) The bar graph represents the mean and the standard deviation of the Comfort scores assessed by the subjects after wearing each device. The red asterisk indicates the sample significantly (*p* < 0.05) different from the other ones, as demonstrated by the Duncan’s post-hoc tests. (**B**) At the end of the experiment the subject had to rank the devices in terms of Comfort, from the most (Position 1) to the less (Position 4) comfortable. The bar graph represents the number of times (frequencies) each device has been ranked in each possible position by the subjects.

In terms of comfort perceived by the subjects wearing the four devices, the BP Gold electrodes obtained the worst scores, significantly lower than those ones obtained by the other electrode types (Duncan test: all *p* < 10^4^, Figure 10A), while no differences emerged among the other three electrode types. In terms of preferences, Quasar and BP Solid electrodes have been more often ranked as the more comfortable device, while the BP Gold as the less comfortable (Figure 10B).

## 4. Discussion

During the last decades great improvements have been made by EEG device manufacturers, so the most recent EEG devices are no larger than a smartphone, thus portable, and equipped with all kinds of key features, such as data storage supports, long-life batteries and wireless connection. At the same time neuroscientific research has produced a great amount of evidences about human brain dynamics and human cognition and behaviors while working or dealing with everydayness activities, showing a great interest in bringing EEG wearable devices within real-life applications [26]. Nevertheless, the EEG devices reliability still lays on recording signals through wet electrodes. Of course, other than some technical issues, such as in particular the skin-electrode impedance instability over time because of gel drying, they constituted a big limitation in terms of usability and comfort. For such a reason, the last decade has been characterized by an increasing interest in EEG dry electrodes, and several types have been developed and proposed, however the scientific community agrees on the fact that wet electrodes are still the gold standard and there is the lack of comprehensive evaluations of these innovative dry electrodes [44]. In this context, the present work aimed at assessing the level of maturity achieved by EEG dry electrodes industry, by comparing three different types, selected from international leading companies, and in order to comprise all the different aspects of the dry electrodes state of art, in terms of material (silicon, gold-coated and solid gel), shape (single or multiple pins), type of contact (resistive or capacitive) and eventual pre-amplification (i.e., active electrodes). Such comparison has been carried on to cover both the aspects of signal quality and device usability, thus providing a very comprehensive approach to the problem.

Before performing the actual analysis comparing the electrodes, the properness of the experimental setup has been validated. In fact, during these experiments the four devices have been tested for each subject in a consecutive way (i.e., not simultaneously). Apart from randomizing the order of the devices testing for each subject in order to prevent any related effect, it was important to verify that actually the brain activity recorded in the same conditions in different moments is comparable. The results obtained by employing the CNTR electrodes, never changed during the whole experiment, confirmed that it is possible to assume a comparable (i.e., it does not significantly change) brain activity during OE and CE rest conditions during the whole experiment, lasting about 1 h and half (Figure 3). This result is crucial for the study, since it allows to compare the different systems assessing that the measured differences are mainly due to the different electrode types and not to the different experimental times.

Regarding the signal quality, the first analysis consisted in analyzing the estimation of the Individual Alpha Frequency. The results (Figure 4) revealed that only Quasar electrodes provided results significantly different from those one obtained through traditional wet electrodes, overestimating the IAF with a MSE of 0.12 Hz^2^. Both the BP gold and the BP solid gel electrodes were able to estimate the IAF in a non-significantly different way from the wet electrodes, with a remarkable MSE of 0.09 Hz^2^ (<1%) related to BP solid gel electrodes. In any case, also the error of Quasar electrodes (<0.2 Hz) could be considered negligible depending on the application. Considering that this parameter, i.e., the IAF, is estimated during Closed Eyes condition, this result does not surprise because of the results obtained in terms of spectra correlation.

In fact, the results in terms of power spectra correlation between dry and wet electrodes highlighted positive high and significant correlations for almost all the comparisons. In particular, during the Open Eyes condition, the overall correlation for all the devices over the frequencies range from 2 to 35 Hz was equal to 0.9 (in terms of R as Pearson’s correlation coefficient). This result is even more remarkable if considering that (i) for a correlation with degrees of freedom equal to 65, as in this case, the R threshold related to a significance level of *p* = 0.00415 (equivalent to *p* = 0.05 corrected for 12 multiple comparisons through Bonferroni method [83]) is equal to R = 0.35; (ii) since the four devices have been recorded in a consecutive (i.e., not simultaneous) way, it is not possible to achieve correlations of 1, as demonstrated by the Control electrodes (Figure 5). With respect to the latter, the mean correlation decreasing is of about the 5%, however the three dry types did not show any significant difference among them. Also, during the Closed Eyes condition, the overall correlation for all the devices over the frequencies range from 2 to 35 Hz was positive, high and significant, with a mean R equal to 0,83 and without any significant difference among the 3 dry types (Figure 5). However, it seems that during Closed Eyes the dry electrodes worked worse than during Open Eyes condition, since the mean correlation decreased from 0.9 to 0.83, anyhow it is important to consider that also the correlation over the Control electrodes decreased from 0.95 to 0.9 (Figure 3). Therefore such decreasing, or at least a part of it, is probably due to a certain difference in terms of mental states (we are assuming a certain similarity among the power spectra between the same conditions recorded in different moments, but it is not possible to quantify it). Also in this case, the results are remarkable if considering that (i) the correlation values are much higher than R = 0.35 (R threshold related to *p* < 0.00415, equivalent to *p* = 0.05 for 12 multiple comparisons); (ii) the mean correlation decreasing with respect to the Control electrodes is of about the 9%, however the three dry types did not show any significant difference among them.

The notable results in terms of correlation are confirmed if considering the single channels and sub-bands (Figure 6 and Figure 7): despite a small decreasing in terms of correlation moving from frontal to parietal sites (probably due to the hairs), significant correlations have been obtained for all the dry types during Open Eyes condition. Only over the F3 site, the BP solid electrodes provided correlation values significantly higher than the other two solutions, while within the lower Beta band the mean correlation between all the dry electrode types and wet electrodes was not significant on average. Also for the Closed Eyes condition, significant correlations have been obtained for all the dry types for each channel and for each sub-band. Only over the P3 site, the Quasar electrodes provided correlation values significantly higher than the other two solutions. Actually, also during Closed Eyes condition a correlation decreasing appeared moving from frontal to parietal sites for BP Gold (−15%) and BP Solid (−16%) electrode types, while Quasar type did not show any particular variation (≈1%). However, it is important to note that the correlation values over the frontal sites for BP Gold and BP solid electrodes were higher than Quasar electrodes. In other words, it seems that Quasar electrodes worked slightly worse over the frontal electrodes, but their performance remained stable over the scalp. On the contrary, BP Gold and BP Solid electrodes worked better over the frontal sites but suffered over the hairy ones. However, it is important to remark that these observations are made just on trends, since non-significant differences arose from statistical analysis.

Finally, in terms of mental states classification, the use of the three dry electrode types was equivalent to the employment of wet electrodes, since statistical analysis did not reveal any significant difference (Figure 8).

Regarding the aspects related to usability, first of all the dry electrodes were able to reduce drastically the time necessary to plug the system: the time of montage of the three dry electrodes were all significantly lower than that one of wet electrodes (Figure 9). In addition, Quasar and BP Solid electrodes allowed to even halve the times of montage of wet electrodes, they were also significantly lower than those one of BP Gold electrodes while did not significantly differ among them. The exponential decreasing of time needed to prepare the EEG system is even more remarkable if considering that, in case of wet systems, it is function of the number of electrodes (i.e., it is the sum of the times to adapt the impedances related to all the electrodes). In the present research no more than 10 EEG channels (including references and ground) have been employed, nevertheless the advantage due to the use of dry technology will become more and more relevant while increasing the number of electrodes. High-resolution EEG [85] are usually based on 64 up to 256 electrodes, in this case reliable dry technology will produce incomparable benefits. Finally, in terms of comfort perceived by the user (Figure 10), the results revealed that the BP Gold electrodes were not as comfortable as the other competitors. If such result could imply that actually wet electrodes are not so annoying, it has to be taken into account that in such evaluation the factor “pain” was more important that the annoyance due to have gel and abrasion over own scalp, probably also because of the few electrodes employed (7). However, in terms of preference (Figure 10B), the subjects used to prefer Quasar and BP Solid electrodes more than wet ones.

Before concluding, it is important to take into account that the electrodes compared within this study have been used jointly with their own acquisition systems, therefore it could be an additional variable influencing the results. It is also true that it is not possible to use these electrodes with whatever EEG device, thus the overall performance (in terms of signal quality) intrinsically depend on both the factors, i.e., the electrodes and the acquisition system.

In conclusion, the dry electrodes employed during this research, selected among the more recent solutions provided by EEG industry, revealed that a very high level of maturity has been reached in this field, both in terms of reliability (i.e., signal quality) and usability. No particular differences arose among them in terms of signal quality, and of course the aim of the research was not to identify a “winner”, however because of the great success achieved by Quasar and BP Solid electrodes also in terms of usability and comfort, it is possible to suggest that solutions based on multiple-pins and soft materials (such as the solid gel) are preferable with respect to the single pin solutions. However, it is possible to claim that actually the gap between wet and dry electrodes is ready to die out, and it is time for literally a “dry revolution” within the EEG research field. This important change would have great scientific and economical consequences, if considering that a recent Marketing Report (RNR Research, [86]) currently estimates an expected market of one Billion USD for Wearable EEG Device with a Compounded Average Growth Rate of 7.13% over the next 10 years. If considering that recently practical dry electrodes have been identified as one of the two disruptive technologies in BCI research [36,37], the results of this research pave the way for a more intensive employment of dry technology within the BCI research and applications. Recent works already validated in highly realistic settings BCI-based systems able to support operators while working [28,87], so in this context the legitimation of dry technology will facilitate the acceptability of BCI solutions within everydayness applications.

The present work does not provide a quantification of morphological signal characteristics, such as signal-to-noise ratio, however the idea at the basis of the present work, accordingly to the recent trends within the BCI domain [26,88,89], is that, despite an unavoidable decreasing in signal quality, it is possible to consider dry systems reliable for non-clinical and goal-oriented applications (e.g., discriminate two different mental states).

Finally, according to the needs highlighted by recent works [44], this work also suggests a comprehensive methodology to evaluate different electrode concepts from different perspectives. The results of the present research appear really impacting for the community but still preliminary, since they were derived from a sample of 12 subjects with “not-too-long” hairs (<8 cm), therefore future works would aim at enlarging the sample size and phenotype as well as integrating these comparative analyses with new dry electrodes concepts.

## 5. Conclusions

The present work aimed at assessing the level of maturity achieved by EEG dry electrodes industry, by comparing three different types with traditional wet electrodes, still considered as gold-standard in terms of signal quality. The results of this study pointed out the great level of quality achieved by dry solutions, since all the tested electrodes were able to guarantee the same quality levels of wet electrodes, allowing at the same time to significantly reduce times of montage and increase users’ comfort. In particular, for the latter aspect solid gel and multiple-spikes solutions overcame the single-pin one. However, in general it is possible to claim that finally it is time for the “dry revolution” within the EEG applied research.
